# Case studies of fifteen novel species successfully aided with the use of a veterinary teletriage service

**DOI:** 10.3389/fvets.2023.1225724

**Published:** 2023-12-05

**Authors:** Shadi J. Ireifej, Justin Krol

**Affiliations:** ^1^VetTriage, Las Vegas, NV, United States; ^2^Aquatic Animal Health Research Unit, United States Department of Agriculture-Agricultural Research Service, Auburn, AL, United States

**Keywords:** telemedicine, telehealth, exotics, wildlife, video session, veterinary, teletriage, teleadvice

## Abstract

The veterinary medical field is constantly growing and evolving. Embracing the growth of readily available video conferencing, and potentially spurred by events such as the COVID-19 pandemic causing the public to seek alternatives to physical contact for medical advice at their local veterinary clinic, the use of long-distance advice or telehealth is a rapidly developing field in its own right. Here we present case studies using a teletriage service, VetTriage, to provide health care advice to clients with underserved species including presenting complications, actions taken during the session, medical advice given, and follow-up of the case when possible. In addition to the everyday difficulty of accessing rapid medical care in recent years, there are households with underserved animal groups such as exotics (small mammals, reptiles, birds, fish, etc.), found wildlife, and in some areas of the country, large animals (horses, cows, etc.). Teletriage services have the potential to reach these underserved animal groups providing a vital service where otherwise no help may be available.

## 1 Introduction

Approximately 57% of U.S. households own pets (1, 2). Where there are over 48 million households owning dogs, over 31 million keeping cats, over 3.5 million having birds, and almost a million owning horses, there are far fewer households that own other small mammals (ferrets, rabbits, etc.), reptiles, fish, and other less commonly known or acquired species^1^. According to Animal Sheltering Pets by the Numbers, in 2017 the number of dogs was estimated at 89.7 million and the number of cats was estimated at 94.2 million^2^. In Great Britain, dogs make up 64.8% of the veterinary-visiting population, with cats, rabbits, and other species making up 30.3, 2.0, and 1.6%, respectively (3). Additionally, pet ownership is highest in more rural states of the United States, making up 65–72% of households each in Wyoming, West Virginia, Nebraska, Vermont, and Idaho^1^. Coinciding with the low numbers of owned non-dog and non-cat small mammals, reptiles, birds, and fish in the United States and Great Britain, and the high number of pets owned overall in more rural regions of the United States, access to veterinary care for those less acquired species in rural demographics becomes difficult. A recent press release shows that pet ownership has increased from an estimated 67% of U.S. households that own a pet to an estimated 70% (4). Within that same study, 14% of the respondents (pet owners and non-pet owners) obtained a new pet during the COVID-19 pandemic (4). The species type and percent acquired that were influenced by the pandemic are as follows: saltwater fish (60%); dogs (47%); birds (46%); small animals (46%); cats (40%); freshwater fish (34%); reptiles (27%); and horses (27%) (4). The majority of U.S. practices are private (60.5%) and either companion animal exclusive (57.9%) or companion animal predominant (15.8%), leaving pet owners of non-dog and non-cat small mammals, reptiles, birds, and fish with limited or without access to local veterinary care (5). As an increasing number of minor species are acquired as pets, the demand for their veterinary care will continue to grow.

In human medicine, access to care in rural areas also poses a challenge with several studies demonstrating the utility of telemedicine to help fill this void. Medical examples where telemedicine improved patient access to care in rural regions include acute pediatric trauma (6), care of ICU patients that otherwise lacked 24-h critical care physician coverage (6, 7), autism assessment and treatment (8), and surgical assessment pre-operatively and post-operatively (9). As telehealth/medicine has been shown to have great benefits in the medical world for humans, its application in veterinary medicine is beginning to be explored, especially for species that are underserved in their region or minor species overall. Here we outline 15 telehealth cases of animal species that are considered either less conventionally owned or with whom telehealth services have not been reported for the purposes of medical triage.

## 2 Materials and methods

### 2.1 Animals reported

Herein, we report 15 novel animal species served with a telehealth service. In addition, the reported cases did not have access to timely or geographically available physical veterinary care. The cases described were triaged with the use of a novel teletriage video platform on an emergency basis by a Doctor of Veterinary Medicine (DVM). We herein report one of each species: squirrel, sugar glider, pleco fish, tortoise, chinchilla, sea otter, pig, ball python, hen, horse, rabbit, goat, duck, gecko, and ferret. All animals were assessed virtually utilizing synchronous video and audio teleconferencing using proprietary software (http://vettriage.com). Teleconferencing was performed using a variety of desktop and mobile electronic devices of the veterinarian's and the client's choosing. Animal data was collected at the time of the triage session. Triage sessions are available 24 h a day, 7 days a week (24/7), with decision-making and medical advice given reflecting the status of physical veterinary locations (open or closed) at the time of start of the session. All owners of animals (clients) enrolled in the study acknowledged the legal and ethical limitations involved with veterinary telehealth prior to engaging in teleconferencing with the U.S.-licensed Doctor of Veterinary Medicine. The disclaimer contained language pertaining to the veterinary–client–patient relationship (VCPR), explaining the current medical, ethical, and legal junctions of the VCPR as they currently stand (2019–present). All animal and pet caretakers requested teleconferencing on their own accord for the purpose of teletriage and not for the purposes of the study. Animals were included in the study if the client acknowledged the legal and ethical limitations in veterinary telehealth, accepted the required fee for the videoconferencing, perceived the animal to be afflicted or potentially afflicted with a medical or traumatic emergency, had good cause to utilize such a service, owned a species of the rarity described, and the teleconferencing was successfully carried out to completion. All sessions were carried out by the same Doctor of Veterinary Medicine (author S.J.I.). Differential diagnoses, where applicable, were discussed with the animal caretakers at the time of the video session. Physical aspects of the animal's condition that are not described were deemed appropriate or normal for that animal during the video teleconferencing. All follow-ups after the teleconference were performed by S.J.I. via both electronic mail and cellular telephone call, with the client response or lack thereof documented.

### 2.2 Platform design

No software download or hardware installation was needed for the telecommunicating video service. The client creates an account on the website http://vettriage.com from desktop or mobile electronic device, follows the directions on the website, acknowledges and electronically signs the disclaimer, purchases the teletriage service via their credit card, debit card, or third-party electronic commerce company, and carries out the teletriage session with the U.S.-licensed DVM, 24/7, regardless of the species and the type of emergency. Online, real-time chat service for all animal/pet owner inquiries, technical support issues, questions, and concerns were available 24/7 on the website http://vettriage.com. Technical support for pet owners was available 24/7 prior to or during any given video session via a dedicated phone line as well. The proprietary software embedded and accessible within the website was made agnostic to any desktop and mobile device, any search engine used, and all internet types and speeds.

In its standard functionality, all sessions on the platform are carried out in a synchronous mode on video by veterinarians on a 24-h basis by employing veterinarians who reside in various time zones adequate for 24-h coverage and who devote themselves to shifts that best fit their personal and professional lifestyles to meet the needs of such coverage.

The proprietary video technology supports H.264 video compression standard. It requires 300 kbps minimum bitrate per stream for video. Video resolution is either 640 × 480 or 1,280 × 720 pixels.

## 3 Case studies

### 3.1 Case #1

A 2-year-old female-intact squirrel, weighing 2.5 lb, presented from Texas with clear nasal discharge, white-colored right ocular discharge, right aural inflammation, sniffling, and occasional sneezing. The squirrel was owned by the client since 1 day of age. The squirrel had no other noted clinical signs. The squirrel had a prior medical history of white-colored ocular discharge that was treated successfully by manually applying an unknown antibiotic ointment purchased online on the affected eye via cotton ball. The client's inquiry was to whether the same or a similar treatment measure could be followed or if other palliative care was needed.

The video examination presented an over-conditioned squirrel with a body condition score 4 out 5 residing in the client's closet on the upper shelf with adequate bedding. The squirrel was bright, alert, and responsive with minimal evidence of ocular, otic, or nasal discharge.

The pet was deemed stable, not requiring immediate emergency evaluation and intervention. The advice included pursuing an in-person veterinary examination with a veterinarian familiar with the species or a wildlife rescue/rehabilitation facility, home monitoring for specific clinical signs consistent with respiratory distress for the presumptive upper respiratory tract infection, conjunctivitis and otitis externa, and airway humidification therapy at home, described as utilization of a humidifier up to three times daily for 10–15 min each time in a small room. Alternative to the humidifier, the client was advised to allow the home bathroom to fill with steam via a hot shower up to three times daily for 10–15 min each time and warned to not place the squirrel in the hot shower and to always accompany the pet during such therapies.

Upon follow-up with the client 1 year and 4 months post-session, the squirrel made a complete recovery solely from the advice given and the condition did not recur. In-person veterinary consultation or in-person wildlife rescue/rehabilitation evaluation was not pursued by the decision of the client.

### 3.2 Case #2

A 5-year-old male-intact sugar glider from the state border of Wisconsin and Minnesota who was acquired the day prior presented with the chief complaint of a possibly infected forehead gland and a lesion on the chest. The previous owners of the pet had reported these conditions in his prior medical history and they was presumed to be attributed to hormonal influence from being an intact male. No other clinical signs were reported. The current owners were cleaning the region with a cotton swab as needed. The client sought additional advice to aid in palliative care for this newly acquired pet.

The video examination revealed a bright, alert, and responsive sugar glider with a ventral sternal swelling that was haired, mildly ulcerative, slightly effusive, spherical in shape, sized 1–2 millimeters in diameter, which was not painful to the sugar glider upon palpation by the pet owner. The forehead lesion appeared as a slightly raised, haired, non-ulcerated swelling that was not painful on palpation and did not produce any discharge.

The pet was deemed stable, not requiring immediate emergency evaluation and intervention. The advice given included an in-person evaluation by a veterinarian familiar with the species for diagnostics, treatment, and castration, the application of an over-the-counter (OTC) triple antibiotic ointment (Bacitracin zinc/Neomycin sulfate/Polymyxin B sulfate) to the affected areas every 8–12 h for 2 weeks or until healed, and monitoring for specific clinical signs consistent with systemic disease and sepsis should the condition progress.

The client was lost to follow-up 18 months post-session.

### 3.3 Case #3

A pleco fish (*Hypostomus plecostomus* or suckermouth catfish) presented from Virginia. The fish had leapt out of its tank while the owner was on a video conferencing meeting at home.

The owner's spouse presumed the fish was dead and threw the pet into the trash. Approximately 5 h after the incident had occurred, the client noted movement in the trash bag and immediately recovered the fish alive. The client placed it back into the tank and then contacted the teletriage service. Prior to the incident, the fish was deemed normal.

The video examination revealed a normal pleco fish with white-colored to hazy eyes and brown-colored dog hairs on its body. The hair originated from thrown-out dog hair from the shedding of the German Shepherd dog at home. The fish appeared to behave normally in the tank, sucking on the glass as expected and the client was able to gently clean the hair off the fish.

The pet was deemed stable, not requiring immediate emergency evaluation and intervention.

The advice given to the client included an in-person evaluation by a veterinarian familiar with the species as soon as possible as well as monitoring of clinical signs associated with continued distress.

Upon follow-up with the client 1 year 5 months post-session, the pleco fish recovered fully and was normal. In-person veterinary consultation was not pursued by the decision of the client.

### 3.4 Case #4

An 8-month-old male intact tortoise of unknown weight presented from California with the chief complaint of excessive weight gain in a short period of time, swollen eyes, urinating frequently, white penile discharge, and a decreased appetite. The clinical signs were non-progressive over a period of 2 weeks. For 1 day during that 2-week period, the owner noted clear ocular discharge that resolved without treatment. The tortoise had been owned his entire life by the same owner. He resided in a fish tank with an environmental tank temperature of 99°F, ranging between 98 and 103°F. The humidity in the tank was not measured. The client had an appointment scheduled 12 days from the date of presentation. The client desired triage and palliative care pending the appointment if deemed appropriate. The video examination revealed an otherwise grossly normal tortoise save for bilaterally swollen eyes.

The pet was deemed sufficiently stable to await that appointment, not requiring immediate emergency evaluation and intervention. The advice given included clinical signs to monitor for the progression of disease, purchasing a humidity measurement tool, and airway humidification therapy at home, consisting of utilizing a humidifier up to three times daily for 10–15 min each time in a small room. Alternatively, the client could also consider allowing the home bathroom to fill with steam via a hot shower up to three times daily for 10–15 min each time. The pet owner was warned not to place the tortoise in the hot shower and to always accompany the pet during such therapies.

The patient was lost to follow-up 3 months post-session.

### 3.5 Case #5

A female-intact chinchilla presented from Alaska for inappetence, left ocular swelling, and left ocular crusting. She had been owned for 3 months by the client. No other clinical signs were reported. The client sought palliative care advice at the time of service.

The video examination depicted a bright, alert, and responsive chinchilla with left ocular blepharospasm, superior eyelid swelling, and facial pruritus. No other overt abnormalities were observed. The pet was deemed stable, not requiring immediate emergency evaluation and intervention. The advice given to the pet owner included an in-person evaluation by a veterinarian familiar with the species within the following 7 days, the application of a warm washcloth compress to the affected region two to three times daily for 10–15 min at a time for the next 3–5 days, ensuring the pet tolerated the treatment and that the temperature of the cloth was comfortable against the client's skin prior to applying it to the chinchilla, and OTC remedies to consider for palliative care. OTC products discussed included GenTeal^®^ Eye Ointment lubricant, a small amount applied to the affected eye every 8 h as needed, or OTC eye care artificial tears, 3–5 drops applied on the affected eye every 8–12 h as needed. In addition, the client would consider purchasing the OTC ocular antimicrobial, Terramycin Ophthalmic Ointment, and a small amount of it to be applied to the affected eye every 8 h as needed until healed.

Upon follow-up with the client, the clinical signs resolved with the advice given 7 days post-session. A blade of grass was flushed out of the eye. The follow-up 9 months post-session with the client confirmed that the chinchilla was healthy. In-person veterinary consultation was not pursued at the discretion of the client.

### 3.6 Case #6

An unknown gender neonatal northern sea otter pup, weighing 5 lb, of <7 days of age presented through a wildlife response team from Alaska to assess for overall stability with plans to transport the otter to the nearest veterinary clinic followed by a 4-h drive to a water taxi.

On video examination, the otter was assessed to be ~5% dehydrated based on oral examination and skin turgor, but otherwise bright, alert, and responsive. The advice given included the administration of subcutaneous fluid therapy prior to the trek to the water taxi. S.J.I. facilitated the identification of the nearest emergency on-call veterinary clinic able and willing to provide subcutaneous fluid therapy for the wildlife rescue personnel and close monitoring prior to the drive. The arrival of the sea otter puppy to the clinic was confirmed successful.

Upon follow-up with the rescue organization 4 months post-session, the sea otter was within normal limits and waiting for permanent residency.

### 3.7 Case #7

A neutered, male, 1.5-year-old pig of unknown body weight presented from Texas for lethargy, anorexia, and the underbelly feeling warm to the touch. No other clinical signs were reported. Prior medical history included a double ear infection and an upper respiratory tract infection, both of which were self-limiting. The client contacted the service to decide if emergency care should be sought.

The video examination by the veterinarian revealed an extremely lethargic pig with minimal response and increased respiratory rate in sternal recumbency. The client was instructed on how to record the temperature using a digital thermometer and he reported a rectal temperature of 106°F. The pet was deemed unstable, requiring immediate emergency evaluation and intervention. Emergency consultation with the nearest DVM familiar with the species was advised.

The client consented, but the outcome is undetermined as the patient was lost to follow-up 8 weeks post-session.

### 3.8 Case #8

A 3-year-old male ball python presented from Ohio for gagging overnight, characterized by being 1 min in duration and associated with whole-body tensioning. He had recently completed a shedding. He was being fed every 2 weeks with two small-sized mice. His last elimination occurred 2 weeks prior when he was also last fed. Other than obesity caused by being fed two rats a week during ownership with a prior owner, there existed no prior medical history. His tank enclosure was 3.5–4 feet in length, and he resided alone. His tank had a humidity ranging from 52 to 70% and was never below a temperature of 70°F. It contained gravel, a heat lamp, and non-toxic plants. He had not been observed to ingest the plants. The owner found skin that morning from shedding. The client sought triage as well as palliative care advice until an appointment with the local veterinarian can be fixed.

The video examination showed a normal snake, save for consistent generalized abdominal pain on palpation by the client. The pet was deemed stable, not requiring immediate emergency evaluation and intervention. The client was advised to consult with an in-person DVM familiar with the species for examination and diagnostics as soon as possible.

Following up with the in-person veterinarian revealed dietary indiscretion from an ingested mouse, triggered by the stress of shedding. He was prescribed three doses of metronidazole and a syringe worth of Bene-Bac^®^ Plus probiotic gel by the in-person veterinarian. Bene-Bac^®^ Plus was given in one gram doses every other day in pairing with the metronidazole. Following that in-person visitation, the client switched from the previous supplier of snake food and had not observed a recurrence of clinical signs since.

A follow-up after 6 months revealed resolution of the clinical signs and the snake doing well.

### 3.9 Case #9

A hen of unknown age and weight presented from Nevada after being attacked by a dog 4 days prior. External wounds were not evident according to the client, but the hen was unable to ambulate. No other clinical signs were noted. The inability to ambulate was non-progressive.

There was no medical history prior to this event and no medications were being administered. The client had decided to not pursue in-person evaluation and sought palliative care.

The video examination revealed a hen with paraparesis of both pelvic limbs with minimal motor function and normal nociception. The remainder of the video examination was unremarkable. The advice given to the client included an in-person evaluation by a veterinarian familiar with the species as soon as possible and to perform physiotherapy as shown in various YouTube videos depicting physiotherapy tutorials typically performed on dogs. The client was also shown how to perform various safe and possibly efficacious physiotherapy techniques that can be used via the video session in real time.

Upon follow-up, the hen had normalized within 1 week follow-up post-session after instituting the advice given. An in-person evaluation was decided against at the discretion of the client.

### 3.10 Case #10

A 7-year-old male thoroughbred horse of unknown weight presented from California with a history of clinical symptoms including a rectal body temperature of 106°F, inappetence, cough, occasional clear ocular discharge, lethargy, and purulent nasal discharge after being acquired from an auction house 10 days prior. He retired from the racetrack 1 year earlier and adopted 4 months prior for pleasure riding. Prior to the video session, the client initiated treatment with UNIPRIM^®^ (Animal Care, NEOGEN) Powder, a combination of trimethoprim and sulfadiazine.

During the video session, the rectal body temperature was 99°F. Further examination revealed a dry, hacking cough but was otherwise unremarkable. The client sought triage to ensure the measures taken thus far were appropriate and assess the need for an in-person visit. The advice given included airway humidification therapy at home, consisting of utilizing a humidifier up to three times daily for 10–15 min each time in a confined enclosure, as well as increasing the frequency of controlled walks to aid in airway movement. In-person visitation was advised within 30 days to confirm a diagnosis and evaluate treatment response adequately.

This patient was lost to follow-up 10-months post-session.

### 3.11 Case #11

A male, 3-year-old rabbit of unknown weight presented from California for an acute onset of diarrhea, severe lethargy, and anorexia of several hours duration. There was no known exposure to toxic substances or foreign bodies. He has been owned for the entirety of his life by the current owners with no prior medical history and no current medications. The clients were interested in triage prior to visiting an emergency veterinary hospital.

The video examination revealed a severely lethargic, non-ambulatory rabbit, with minimal responsiveness, and acute abdominal pain on palpation by the client as directed by S.J.I.

Emergency consultation with the nearest DVM familiar with the species was advised. Approximately 28 min after cession of the video session, via e-mail correspondence, the rabbit appeared to be ambulatory with a sudden burst of energy according to the client. The client questioned whether visiting the nearest emergency hospital familiar with the species was warranted. The previous advice given by S.J.I. was reinforced. This patient was lost to follow-up 12 months post-session.

### 3.12 Case #12

A 16-year-old, spayed, female goat of unknown weight presented from Virginia for a 4-day history of abdominal distention and progressive lethargy. The client was administering half a cup of mineral oil mixed with a teaspoon of baking soda orally via a syringe based on her online search to treat what she anticipated to be a bloat. The client also owned a cow and a horse, both of which had no clinical signs. The day prior to the presentation, the client administered 2 mL of Clostridium Perfringens C & D Antitoxin and a 4-year past expired course of BANAMINE^®^ (flunixin meglumine injection) 2 cc/100 lb, both administered intramuscularly. Urination and defecation were normal. The goat did ingest the horse's feed consisting of timothy pellets and warm water prior. The goat also was seen to ingest weeds growing from the barn. There were no available veterinarians in the area familiar with the species at the time of the session according to the client.

The video examination revealed obvious flatulence, burping, and severe left-sided abdominal distention. In-person emergency consultation with the nearest Doctor of Veterinary Medicine familiar with the species was advised. Other advice given included administration of flunixin meglumine, Gas-X (https://www.gas-x.com), Simethicone Emulsion (https://www.vetsfarma.com), and increasing the frequency of controlled walks to aid in gastrointestinal motility.

This patient was lost to follow-up 12 months post-session.

### 3.13 Case #13

A female duck of unknown age and weight presented from Illinois for an acute onset of lethargy and not walking with the other ducks in her coop that morning. According to the client, the duck held her beak to the ground and her tail down. Her appetite status was unknown, and she was drinking less water than normal. Her droppings were grossly normal. There were no other reported clinical signs, and the other ducks have no noticeable abnormalities. She was allowed to roam freely outdoors but no known toxic exposures were observed to be ingested. There were no available veterinarians in the area familiar with the species at the time of the session according to the client.

The video examination revealed abnormalities consistent with the owner's description of the duck. She exhibited a normal gait, was aware of her surroundings, but noticeably lethargic, carrying her head and tail down, and preferred to ambulate on her own, away from the raft.

In-person emergency consultation with the nearest DVM familiar with the species was advised. The advice given included providing enhanced water to maintain hydration: 8 teaspoons of granulated sugar, 1/2 teaspoon of sea salt, 1/2 teaspoon of baking soda, 1 gallon of water with one-third of the water replaced with unflavored Pedialyte, but to avoid daily addition of vitamins and electrolytes to prevent diarrhea. The client was advised to maintain an increased ambient temperature, to seek out a local Tractor Supply Company store for OTC antimicrobials (antibiotic medications: erythromycin, oxytetracycline, sulfonamides, sulfonamide-trimethoprim, fluoroquinolone, macrolides), and to quarantine her from the raft during the healing period.

This patient was lost to follow-up 8 weeks post-session.

### 3.14 Case #14

A female Leopard Gecko of unknown weight presented from Massachusetts (Martha's Vineyard Island) for the presence of blood at the anatomical location where the tail and pelvic limbs joined, presumably the location of the cloaca, according to the client. The gecko was purchased from a pet store 3 months prior and was judged to be 3–5 years of age at the time. Suspecting a cloacal prolapse, the owner applied sugar water for 15 min and the hemorrhage ceased. No prior medical history was reported, and no current medications or supplements were being administered by the client. There were no other reported clinical signs.

The video examination revealed no abnormalities. Monitoring was advised until they were able to schedule an appointment with their in-person veterinarian for further evaluation.

As of follow-up 11 days post-session, the patient showed continued improvement but acutely perished due to hypothermia (winter months) while in transit on a ferry to the veterinarian for a follow-up in-person evaluation. The owner reported that the clinical sign resolved and did not recur after the video teleconferencing session was completed.

### 3.15 Case #15

A 1-year-old, standard, petite-sized female ferret of unknown weight presented from New York. The locally available veterinarians were unfamiliar with the species on discussion with the client. The chief complaints were a few days history of decreased appetite, severe lethargy, and drinking less water. Urination had been normal, but she had been defecating less with the feces possibly being darker in color and less solid than normal. There were no other clinical signs noted.

The video examination revealed overt lethargy and weakness. In-person emergency consultation with the nearest DVM familiar with the species was advised. The advice given included methods to maintain normoglycemia with over-the-counter products like honey, peanut butter, or other favorable, high caloric supplement commercial products while emergency in-person veterinary care was sought.

This patient was lost to follow-up 4 weeks post-session.

## 4 Discussion

One of the major benefits of telemedicine is its versatility. Each of the aforementioned cases demonstrated how teletriage was performed successfully with uncommonly owned or cared for species and for different purposes as depicted by the clients. It is interesting to note that of the 15 cases reported here, 47% were available on follow-up and the remainder were lost to follow-up. Of the cases on which follow-up information was available, 28.5% sought out in-person evaluation, 14.2% tried but failed, and the remainder did not seek an in-person evaluation. The most commonly reported reason for owners not following up with a traditional care veterinarian was financial limitation (10). Other published benefits of employing telemedicine in veterinary medicine include client convenience (11), poor travel conditions (distance, inclement weather, driving at night), patient stress or anxiety reduction (11, 12), and decreased access to veterinary care due to veterinary shortages (5, 13).

All the sessions reported here occurred successfully and went on to completion with no client experiencing technical difficulties or other technologically related challenges. In one study that evaluated owner satisfaction with the use of videoconferencing for recheck examinations following routine surgical sterilization in dogs, once the dog owners chose videoconferencing, 100% of the owners agreed or strongly agreed that they felt comfortable using the technology and 94% reported that they agreed or strongly agreed that they would be willing to use the telemedicine application again (11).

## 5 Conclusion/clinical relevance

In conclusion, this is the first publication to our knowledge of a squirrel, sugar glider, pleco fish, tortoise, chinchilla, sea otter, pig, ball python, hen, horse, rabbit, goat, duck, gecko, or ferret being successfully assessed and triaged through a novel veterinary telehealth video platform. We acknowledge the limitations of this study such as a smaller sample size, loss of some patients to follow-up, and the challenges of obtaining an accurate assessment through video or audio. Nevertheless, we our study has demonstrated the usefulness of this developing technology, and as the sample size and technology grow, the utility of this kind of service will be further evaluated. It is expected that this modality will become further popular and widely used by pet owners, wildlife rescue personnel, and the veterinary community in the years that follow. When taking into consideration the COVID-19 pandemic, the rise in pet adoptions, the limited access to veterinary care for general practice, specialty and emergency care, and the veterinary staffing shortage, the usefulness and versatility of this novel synchronous video teletriage platform is very clear.

Further studies are needed to explore a larger number of cases of less published species as well as prospective studies evaluating the correlation of the assessment concluded by the veterinarian on such platforms and case outcomes.

## Data availability statement

The original contributions presented in the study are included in the article/supplementary material, further inquiries can be directed to the corresponding author.

## Author contributions

SI is the DVM that provided service for all cases discussed herein and provided initial drafting of the manuscript. JK provided drafts and critical revisions of the manuscript. All authors contributed to the article and approved the submitted version.

## Footnotes

^1^ https://www.avma.org/resources-tools/reports-statistics/us-pet-ownership-statistics

^2^*Animal Sheltering Pets by the Numbers*. Available online: https://www.animalsheltering.org/page/pets-by-the-numbers (accessed February 19, 2020).
